# Clinical profile analysis and nomogram for predicting in-hospital mortality among elderly severe community-acquired pneumonia patients with comorbid cardiovascular disease: a retrospective cohort study

**DOI:** 10.1186/s12890-022-02113-9

**Published:** 2022-08-13

**Authors:** Linjing Gong, Dingxiu He, Dong Huang, Zhenru Wu, Yujun Shi, Zongan Liang

**Affiliations:** 1 Department of Respiratory and Critical Care Medicine, West China Hospital, Sichuan University, No. 37 Guoxue Alley, Chengdu, 610041 Sichuan China; 2 Laboratory of Pathology, Key Laboratory of Transplant Engineering and Immunology, NHC, West China Hospital, Sichuan University, No. 37 Guoxue Alley, Chengdu, 610041 Sichuan China; 3 Department of Emergency Medicine, The People’s Hospital of Deyang, Deyang, Sichuan China

**Keywords:** Severe community-acquired pneumonia (SCAP), Comorbid cardiovascular disease (CVD), The elderly, Nomogram, In-hospital mortality

## Abstract

**Background:**

Researchers have linked cardiovascular disease (CVD) with advancing age; however, how it drives disease progression in elderly severe community acquired pneumonia (SCAP) patients is still unclear. This study aims to identify leading risk predictors of in-hospital mortality in elderly SCAP patients with CVD, and construct a comprehensive nomogram for providing personalized prediction.

**Patients and methods:**

The study retrospectively enrolled 2365 elderly patients identified SCAP. Among them, 413 patients were found to have CVD. The LASSO regression and multivariate logistic regression analysis were utilized to select potential predictors of in-hospital mortality in elderly SCAP patients with CVD. By incorporating these features, a nomogram was then developed and subjected to internal validations. Discrimination, calibration, and clinical use of the nomogram were assessed via C-index, calibration curve analysis, and decision plot.

**Results:**

Compared with patients without CVD, elderly SCAP patients with CVD had a significant poor outcome. Further analysis of the CVD population identified 7 independent risk factors for in-hospital mortality in elderly SCAP patients, including age, the use of vasopressor, numbers of primary symptoms, body temperature, monocyte, CRP and NLR. The nomogram model incorporated these 7 predictors showed sufficient predictive accuracy, with the C-index of 0.800 (95% CI 0.758–0.842). High C-index value of 0.781 was obtained in the internal validation via bootstrapping validation. Moreover, the calibration curve indicative a good consistency of risk prediction, and the decision curve manifested that the nomogram had good overall net benefits.

**Conclusion:**

An integrated nomogram was developed to facilitate the personalized prediction of in-hospital mortality in elderly SCAP patients with CVD.

## Introduction

Community acquired pneumonia (CAP), an important public health problem, is a leading infectious cause of hospitalization and death among adults worldwide [1, 2]. It has been reported that about 21% of hospitalized CAP patients found require intensive care unit (ICU) admission, with 26% of them needing mechanical ventilation, on a basis of surveillance study with a large population. Severe CAP (SCAP) remains a substantial morbidity and mortality, which is caused by exacerbation of lung tissue inflammation. Despite recent advance in antibiotic therapy [3], the mortality of hospitalized SCAP patients still ranges from 12  to  50% [4]. Because SCAP is still a major challenge in ICU, performing interventional and randomized controlled trials (RCTs) in this subgroup of patients could be difficult [5]. Thus, clinicians are supposed to develop a reliable evaluation system to assess the severity of SCAP to take medical interventions timely and prevent the condition becomes worst.

Recent studies showed that the proportion of elderly patients in the ICUs was increasing, however, the prediction of prognosis in elderly patients was quite difficult [6]. Some hospitalized SCAP patients, especially for elderly patients with multiple complications, died within a short period of time after admitting to ICUs [5]. Cardiovascular diseases (CVDs) are the leading cause of death worldwide, representing 31% of all deaths worldwide and 40% of deaths in the Chinese population [7]. CVDs consist of disorders of the blood vessels and heart, including coronary artery disease, cerebrovascular disease, aortic disease, peripheral vascular disease (PVD), and heart failure. In adults, an increased incidence of cardiovascular diseases (CVDs) has been found in those with SCAP, especially the middle-aged and elderly persons [8]. In addition, it was also indicated that SCAP patients with comorbid cardiovascular disease (CVD) exhibited a poor prognosis compared to those without CVD [9]. Due to lack of relevant research, we know little about the clinical characteristics of this population.

Severity assessment is an essential component of the prognostic prediction for CAP patients. To date, there is no consensus in clinical practice on the optimal assessment tools for predicting outcomes in patients with SCAP [3]. Although prognostic prediction scores for pneumonia such as pneumonia severity index (PSI) and CURB-65 scores are widely used [5], there were a few studies in elderly SCAP patients. Some clinical evidence indicated that PSI and CURB-65 may not be reliable evaluation tool in elderly patients [6]. This reflects the strong influence of age, various organ dysfunctions and multitudinous comorbidities on both scoring systems [3]. Based on these observations, it is necessary to develop an effective evaluation system in elderly SACP patients with comorbid CVDs for early identification of potential risk factors in this population.

In the present study, we collected data from patients (aged ≥ 65 years) who were hospitalized due to SCAP, and the associations between CVDs and prognosis of SCAP were explored. A nomogram for predicting the risk of hospital mortality in elderly SCAP patients with comorbid CVDs was also constructed to support clinicians in their therapeutic strategies.

## Materials and methods

### Study population

We retrospectively recruited consecutive patients who diagnosed of SCAP between September 2011 and September 2019 in a 172-bed medical intensive care unit (ICU) of a large tertiary care teaching hospital in Sichuan Province, China. The study was performed in accordance with the declaration of Helsinki and was approved by ethic committee of the West China Hospital of Sichuan University (No. 2021-828). Written informed consent was waived due to the retrospective noninterventional design.

SCAP was defined as pneumonia that acquired outside of the hospital or less than 48 h after hospital admission, fulfilled three or more of the following criteria: respiratory rate greater than or equal to 30 breaths/min, PaO_2_ /FiO_2_ ratio less than or equal to 250 mmHg, new onset mental confusion, WBC count < 4000 cells/mm^3^ (no other causes), platelet count < 100,000 cells/mm^3^, blood urea nitrogen (BUN) greater than 20 mg/dL, core temperature < 36 °C, hypotension with the need of fluid resuscitation, or radiographic findings of new pulmonary infiltrate(s) consistent with CAP diagnosis. Moreover, patients with pneumonia of sufficient severity meeting at least one of the severity criteria, such as receiving mechanical ventilatory support or septic shock with the need for vasopressors even after adequate fluid resuscitation were also included in the study.

The exclusion criteria of this study were as follows: (1) under 65 years old; (2) being pregnant or perinatal; (3) severe immunosuppression: autoimmune diseases, human immunodeficiency virus infection, chemotherapy, or other immunosuppressive therapy; (4) repeated admission; (5) hospital acquired pneumonia; (6) discharged within 24 h of admission; and (7) having incomplete data.

If the patient was admitted multiple times during the study period, only the first admission was included. The diagnosis of CVDs was based on medical records according to the international classification of diseases 10: codes I00–I99. Major adverse cardiovascular events (MACE) included myocardial infarction (MI), arrhythmia, cardiac failure (CHF) and stroke [10]. The occurrence of MACE was diagnosed by the physician in charge. All patients received standard care at the discretion of the physician in charge.

### Data collection

Demographic and clinical variables including age, gender, vital signs on the day of SCAP diagnosis, as well as the comorbidities (cancer, diabetes, chronic hepatic diseases, etc.) were extracted from the institutional electronic medical records. The PSI and CURB-65 scores were determined on the day of SCAP diagnosis. We also extracted the worst value of biological data (hematological data, biochemical parameters, coagulation indicators, etc.) within 24 h of SCAP onset (Table 1). Two trained respiratory clinicians reviewed the medical records and completed the data collection independently, and disagreement was decided by consensus after team discussion.

**Table 1:** Comparisons of clinical characteristics and outcomes among elderly SCAP patients

Variables	Total (n = 2365)	nonCVD-SCAP (n = 1952)	CVD-SCAP (n = 413)	*P*-value
Demographic characteristics				
Age (years old)	75.30 (75.01–75.60)	75.09 (74.77–75.41)	76.31 (75.59–77.03)	**0.002**
Male, n (%)	1529 (64.7)	1266 (64.9)	263 (63.7)	0.650
Treatment				
IMV, n (%)	2343 (99.1)	1935 (99.1)	408 (98.8)	0.513
Vasopressor, n (%)	1432 (60.5)	1139 (58.4)	293 (70.9)	**0.000**
Primary symptoms				
Fever, n (%)	506 (21.4)	418 (21.4)	88 (21.3)	0.962
Cough, n (%)	736 (31.1)	538 (27.6)	198 (47.9)	**0.000**
Sputum, n (%)	276 (11.7)	216 (11.1)	60 (14.5)	**0.046**
Dyspnea, n (%)	420 (17.8)	336 (17.2)	84 (20.3)	0.131
Insanity, n (%)	32 (1.4)	26 (1.3)	6 (1.5)	0.847
Chest pain, n (%)	50 (2.1)	36 (1.8)	14 (3.3)	**0.047**
Comorbidities				
Cancer, n (%)	428 (18.1)	368 (18.9)	60 (14.5)	**0.038**
Diabetes mellitus, n (%)	392 (16.6)	260 (13.3)	132 (32.0)	**0.000**
Dementia (%)	37 (1.6)	22 (1.1)	15 (3.6)	**0.000**
Chronic hepatic diseases (%)	65 (2.7)	54 (2.8)	11 (2.7)	0.907
Chronic renal diseases (%)	175 (7.4)	113 (5.8)	62 (15.0)	**0.000**
Chronic pulmonary diseases (%)	613 (25.9)	454 (23.3)	159 (38.5)	**0.000**
Vital signs on admission				
Respiratory rate (breath/min)	19 (14–23)	18 (14–23)	19 (14–24)	0.297
Heart rate (beat/min)	94 (78–110)	93 (78–110)	97 (80.5–112)	**0.006**
Systolic blood pressure (mmHg)	131 (110–149)	131 (111–150)	129 (110–147)	**0.043**
Diastolic blood pressure (mmHg)	70 (59–81)	70 (59–81)	70 (59–81)	0.577
Temperature (°C)	36.5 (36.3–37.0)	36.5 (36.3–37.0)	36.5 (36.2–37.0)	0.215
SPO_2_ (%)	100 (98–100)	100 (98–100)	100 (97–100)	0.361
PaO_2_/FIO_2_	183.11 (122–241.14)	186.37 (126.13–242.06)	169 (106.58–223.25)	**0.013**
Laboratory examinations				
White blood cell (× 10^9^/L)	9.57 (6.58–13.37)	9.49 (6.51–13.22)	10.09 (6.84–14.15)	0.063
Monocyte (× 10^9^/L)	0.43 (0.27–0.62)	0.43 (0.27–0.62)	0.44 (0.24–0.63)	0.949
Neutrophil (× 10^9^/L)	7.87 (4.91–11.53)	7.69 (4.8725–11.33)	8.47 (5.13–12.59)	**0.015**
Lymphocyte (× 10^9^/L)	0.83 (0.53–1.24)	0.85 (0.54–1.25)	0.78 (0.48–1.16)	**0.026**
Platelet (× 10^9^/L)	165 (106–237)	165 (108–239)	165 (95–230.5)	0.203
Hemoglobin (g/L)	108 (89–127)	108 (89–127)	109 (88–126)	0.919
Albumin (g/L)	32.2 (28.2–37.5)	32.4 (28.5–37.6)	31.2 (27.3–37.05)	**0.005**
Globulin (g/L)	25.4 (21.7–29.0)	25.4 (21.83–28.9)	25.3 (21.5–29.2)	0.679
ALT (IU/L)	20 (12–37)	19.5 (12–36)	20 (13–39)	0.433
AST (IU/L)	27 (19–47)	27 (19–47)	29 (20–48.5)	0.243
Total bilirubin (µmol/L)	5.3 (3.5–8.4)	5.3 (3.5–8.38)	5.2 (3.5–8.4)	0.977
Direct bilirubin (µmol/L)	11.2 (7.8–16.55)	11.3 (7.8–16.58)	10.8 (7.9–16.6)	0.565
APTT (s)	32.3 (27.9–38.9)	32.15 (27.9–38.4)	33.3 (28.2–40.85)	0.078
PT (s)	12.9 (11.8–14.4)	12.9 (11.9–14.4)	13.1 (11.8–14.6)	0.591
Fibrinogen (g/L)	3.53 (2.64–4.61)	3.53 (2.63–4.61)	3.58 (2.72–4.62)	0.531
D-dimer (mg/L)	4.19 (2.23–9.05)	4.19 (2.20–8.98)	4.19 (2.37–9.35)	0.304
Creatinine (µmol/L)	75 (56.85–109)	75 (56–109)	78 (58–112)	0.156
Uric acid (µmol/L)	243 (152.9–346.65)	241.7 (153.775–340.53)	248 (149.9–364)	0.36
Lactate (mmol/L)	1.5 (1.1–2.0)	1.5 (1.1–2)	1.5 (1.1–2.1)	0.109
Troponin T (ng/mL)	27.9 (19.95–68.8)	27.45 (19.6–67.25)	30.4 (21.45–81.65)	**0.023**
BNP (pg/mL)	1078 (553.5–3939.5)	1037.5 (524.5–3842.5)	1195 (691.5–4661)	**0.048**
BUN (mg/dL)	7.63 (5.36–12.2)	7.60 (5.3–12.00)	7.77 (5.7–13)	0.077
Glucose (mmol/L)	7.12 (5.88–9.59)	7.09 (5.86–9.53)	7.34 (5.99–9.94)	0.318
CRP (mg/L)	63.45 (27.55–105)	63.45 (27.03–104)	63.45 (30.85–107.50)	0.431
PCT (ng/mL)	0.33 (0.15–1.10)	0.33 (0.14–1.05)	0.33 (0.18–1.34)	0.063
Major adverse cardiovascular events				
Myocardial infarction, n (%)	48 (2.0)	32 (1.6)	16 (3.9)	**0.003**
Arrhythmia, n (%)	250 (10.6)	197 (10.1)	53 (12.8)	0.100
Cardiac failure, n (%)	120 (5.1)	99 (5.1)	21 (5.1)	0.991
Stroke, n (%)	143 (6.0)	114 (5.8)	29 (7.0)	0.360
Prognosis				
7-day mortality, n (%)	164 (6.9)	114 (5.8)	50 (12.1)	**0.000**
14-day mortality, n (%)	323 (13.7)	235 (12.0)	88 (21.3)	**0.000**
28-day mortality, n (%)	633 (26.8)	461 (23.6)	172 (41.6)	**0.000**
ICU mortality, n (%)	755 (31.9)	560 (28.7)	195 (47.2)	**0.000**
In hospital mortality, n (%)	880 (37.2)	660 (33.8)	220 (53.3)	**0.000**

*p* value of ≤ 0.05 was considered to be statistically significant and shown in bold

ALT, alanine aminotransferase; APTT, activated partial thromboplastin time; AST, aspartate aminotransferase; BNP, brain natriuretic peptide; BUN, blood urea nitrogen; CRP, C-reactive protein; IMV, invasive mechanical ventilation; PCT, procalcitonin; PT, prothrombin time

### Clinical outcome

Patients were followed up until hospital discharge. The primary outcome was in-hospital mortality, and the secondary outcomes were ICU mortality, and 7-day, 14-day and 28-day mortality after the diagnosis with SCAP.

### Statistical analysis

All statistical analyses and graphs were performed using the SPSS for Windows (Version 25.0, Chicago, IL, USA) and R software (Version 4.1.1, https://www.R-project.org/). Continuous variables were expressed as median [interquartile range (IQR), 25–75%] or mean ± standard deviation (SD), while categorical variables were expressed as frequencies (%). The demographic and clinical characteristics of patients were compared between the elderly patients with or without comorbid cardiovascular disease using Student’s t test, Mann–Whitney U test, or chi-square test, as appropriate.

Univariate logistic regression analysis was firstly performed to detect the variables that might be associated with in-hospital mortality of elderly SCAP patients with CVD. The results were presented as an OR with 95% CI. Previous studies have shown that the logistic least absolute shrinkage and selection operator (LASSO) model can actively select from a large and potentially multicollinear set of variables in the regression, resulting in a more pivotal and interpretable set of risk factors [11]. Hence, the LASSO regression and multivariate logistic regression analysis were further utilized to filter possible risk factors from variables mentioned in Table 2. To obtain the optimal predictive features in this study, the lambda in the LASSO regression model was selected by using fivefold cross-validation via minimum criteria and features with nonzero coefficients were collected. The multivariable logistic regression analysis was used to build a predicting model by incorporating the features selected in the LASSO model. Continuous variables were categorized by the cutoff value determined empirically (upper limit or lower limit of the normal range) or by the maximum Youden index. The features were picked through *P*-value, odds ratio (OR) and 95% confidence interval (CI). Finally, by introducing all the selected features, a nomogram prediction model was constructed using R software with the rms package.

**Table 2:** The risk factors for in-hospital mortality using univariate logistic regression analysis among elderly SCAP patients with CVD

Variables	Survival (n = 193)	Dead (n = 220)	OR (95%CI)	P value
Age (year)				
< 76	111 (57.5)	89 (40.5)	Ref	
≥ 76	82 (42.5)	131 (59.5)	1.992 (1.346–2.950)	**< 0.001**
Sex				
Female	70 (36.3)	80 (36.4)	Ref	
Male	123 (63.7)	140 (63.6)	0.996 (0.666–1.489)	0.984
The use of vasopressor				
No	86 (44.6)	34 (15.5)	Ref	
Yes	107 (55.4)	186 (84.5)	4.397 (2.768–6.985)	**< 0.001**
Number of comorbidities				
0	63 (32.6)	25 (11.4)	Ref	
1	49 (25.4)	57 (25.9)	2.931 (1.608–5.343)	**< 0.001**
≥ 2	81 (42.0)	138 (62.7)	4.293 (2.506–7.356)	**< 0.001**
MACE				
No	166 (86.0)	172 (78.2)	Ref	
Yes	27 (14.0)	48 (21.8)	1.716 (1.023–2.879)	**0.041**
PaO_2_/FiO_2_				
≥ 200	89 (46.1)	72 (32.7)	Ref	
< 200	104 (53.9)	148 (67.3)	1.759 (1.180–2.622)	**0.006**
Respiratory rate (breath/min)				
10–19	109 (56.5)	100 (45.5)	Ref	
≥ 20	84 (43.5)	120 (54.5)	1.557 (1.055–2.298)	**0.026**
Heart rate (beat/min)				
< 100	120 (62.2)	109 (49.5)	Ref	
≥ 100	73 (37.8)	111 (50.5)	1.674 (1.130–2.480)	**0.010**
Systolic blood pressure (mmHg)				
90–139	107 (55.4)	135 (61.4)	Ref	
< 90	15 (7.8)	23 (10.4)	1.215 (0.605–2.443)	0.584
≥ 140	71 (36.8)	62 (28.2)	0.692 (0.453–1.058)	0.090
Diastolic blood pressure (mmHg)				
60–89	123 (63.7)	132 (60)	Ref	
< 60	43 (22.3)	66 (30)	1.430 (0.907–2.256)	0.124
≥ 90	27 (14.0)	22 (10)	0.759 (0.411–1.403)	0.379
Temperature (°C)				
< 36.1	28 (14.5)	48 (21.8)	Ref	
≥ 36.1	165 (85.5)	172 (78.2)	0.608 (0.364–1.015)	0.057
Albumin (g/L)				
≥ 35	80 (41.5)	49 (22.3)	Ref	
< 35	113 (58.5)	171 (77.7)	2.471 (1.611–3.789)	**< 0.001**
A/G				
≥ 1.5	61 (31.6)	54 (24.5)	Ref	
< 1.5	132 (68.4)	166 (75.5)	1.421 (0.923–2.188)	0.111
Total bilirubin (µmol/L)				
< 17	144 (74.6)	169 (76.8)	Ref	
≥ 17	49 (25.4)	51 (23.2)	0.887 (0.565–1.392)	0.602
Direct bilirubin (µmol/L)				
< 6.8	131 (67.9)	132 (60)	Ref	
≥ 6.8	62 (32.1)	88 (40)	1.409 (0.939–2.112)	0.097
ALT (IU/L)				
< 40	153 (79.3)	159 (72.3)	Ref	
≥ 40	40 (20.7)	61 (27.7)	1.467 (0.930–2.316)	0.100
AST (IU/L)				
< 36	132 (68.4)	119 (54.1)	Ref	
≥ 36	61 (31.6)	101 (45.9)	1.837 (1.227–2.748)	**0.003**
White blood cell (× 10^9^/L)				
< 10	103 (53.4)	102 (46.4)	Ref	
≥ 10	90 (46.6)	118 (53.6)	1.324 (0.899–1.951)	0.156
Monocyte (× 10^9^/L)				
≥ 0.8	30 (15.5)	21(9.5)	Ref	
< 0.2	25 (13.0)	53 (24.2)	3.029 (1.455–6.303)	**0.003**
0.2–0.49	78 (40.4)	87 (39.5)	1.593 (0.844–3.010)	0.151
0.5–0.79	60 (31.1)	59 (26.8)	1.405 (0.724–2.727)	0.315
Hemoglobin (g/L)				
≥ 90	154 (79.8)	153 (69.5)	Ref	
< 90	39 (20.2)	67 (30.5)	1.729 (1.098–2.722)	**0.018**
Platelet (× 10^9^/L)				
≥ 100	161 (83.4)	140 (63.6)	Ref	
< 100	32 (16.6)	80 (36.4)	2.875 (1.800–4.592)	**< 0.001**
APTT (s)				
< 37	142 (73.6)	126 (57.3)	Ref	
≥ 37	51 (26.4)	94 (42.7)	2.077 (1.369–3.151)	**0.001**
PT (s)				
< 13	112 (58.0)	88 (40)	Ref	
≥ 13	81 (42.0)	132 (60)	2.074 (1.400–3.073)	**< 0.001**
Fibrinogen (g/L)				
< 2	24 (12.4)	29 (13.2)	Ref	
≥ 2	169 (87.6)	191 (86.8)	0.935 (0.524–1.669)	0.821
D-dimer (mg/L)				
< 4	81 (42.0)	98 (44.5)	Ref	
≥ 4	112 (58.0)	122 (55.5)	0.900 (0.609–1.330)	0.598
Creatinine (µmol/L)				
< 100	150 (77.7)	135 (61.4)	Ref	
≥ 100	43 (22.3)	85 (38.6)	2.196 (1.423–3.391)	**< 0.001**
BUN (mg/dL)				
< 10	148 (76.7)	115 (52.3)	Ref	
≥ 10	45 (23.3)	105 (47.7)	3.003 (1.962–4.597)	**< 0.001**
Troponin T (ng/mL)				
< 100	169 (87.6)	161 (73.2)	Ref	
≥ 100	24 (12.4)	59 (26.8)	2.580 (1.532–4.346)	**< 0.001**
BNP (pg/mL)				
< 3364.5	160 (82.9)	128 (58.2)	Ref	
≥ 3364.5	33 (17.1)	92 (41.8)	3.485 (2.199–5.524)	**< 0.001**
Glucose (mmol/L)				
< 7.4	116 (60.1)	94 (42.7)	Ref	
≥ 7.4	77 (39.9)	126 (57.3)	2.019 (1.363–2.991)	**< 0.001**
Lactate (mmol/L)				
< 2.1	152 (78.8)	149 (67.7)	Ref	
≥ 2.1	41 (21.2)	71 (32.3)	1.767 (1.131–2.759)	**0.012**
PCT (ng/mL)				
< 0.5	134 (69.4)	105 (47.7)	Ref	
0.5–1.9	38 (19.7)	59 (26.8)	1.981 (1.225–3.206)	**0.005**
≥ 2	21 (10.9)	56 (25.5)	3.403 (1.938–5.975)	**< 0.001**
NLR				
< 6.54	76 (39.4)	42 (19.1)	Ref	
≥ 6.54	117 (60.6)	178 (80.9)	2.753 (1.767–4.288)	**< 0.001**
CRP (mg/L)				
< 98	155 (80.3)	136 (61.8)	Ref	
≥ 98	38 (19.7)	84 (38.2)	2.519 (1.611–3.939)	**< 0.001**

*p* value of ≤ 0.05 was considered to be statistically significant and shown in bold

Calibration curves were plotted to visually assess how close the nomogram predicted risk is to the actual risk. In order to quantify the predictive performance of nomogram, Harrell’s C-index was measured. the C-index for internal validation was calculated by using bootstrapping (1000 bootstrap resamples). To determine the clinical practicability of nomogram, decision curve analysis was depicted based on the net benefit according to different threshold probabilities in the elderly SCAP patients with comorbid cardiovascular disease. All reported *p* values were two-tailed, and a *p* value of ≤ 0.05 was considered to be statistically significant.

## Results

### Clinical characteristics of elderly SCAP patients

**Fig. 1:**
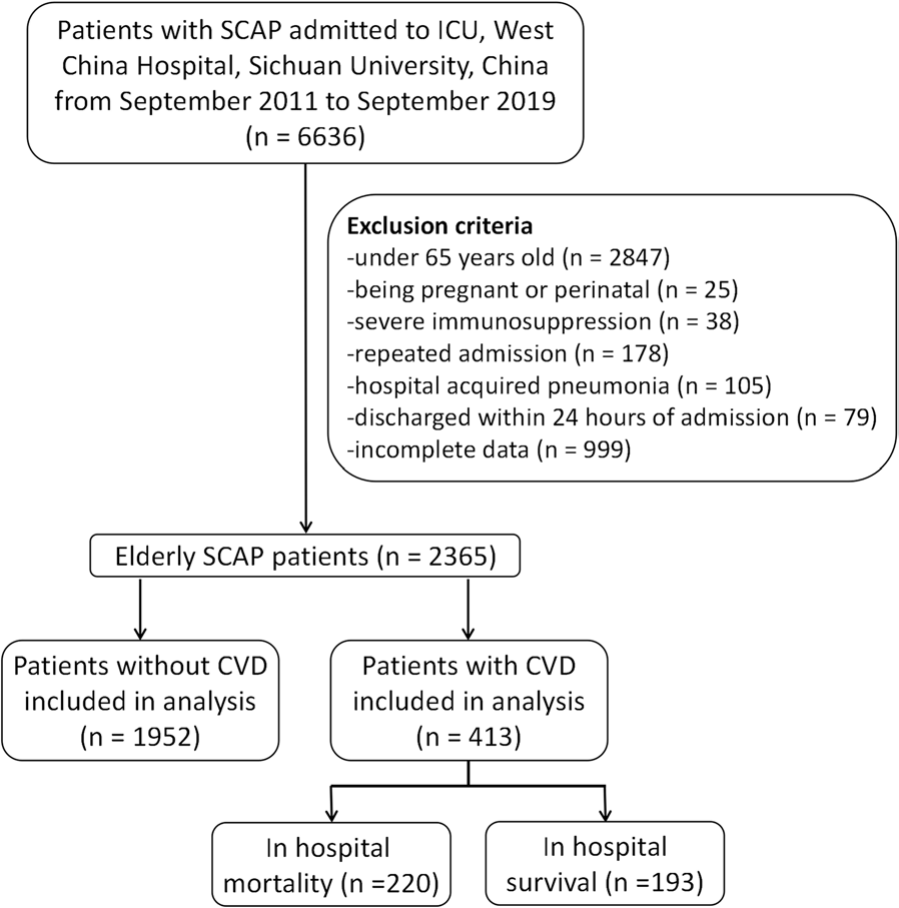
Flow chart of the enrollment of patients in this study

A total of 2365 elderly patients identified SCAP were finally enrolled in the present study according to the inclusion and exclusion criteria. Among them, 413 (17.5%) patients were found to have CVD (Fig. 1). Compared with patients without CVD, the average age and the utilization rate of vasopressor (70.9 vs. 58.4%, p < 0.001) were higher in patients with CVD. As shown in Table 1, CVD patients were more likely to have diabetes mellitus, chronic renal diseases and chronic pulmonary diseases. Besides, they were also more likely to cough (47.9 vs. 27.6%, p < 0.001), sputum (14.5 vs. 11.1%, p = 0.046), as well as to have chest pain (3.3 vs. 1.8%, p = 0.047). When comparing vital signs on admission, patients with CVD were seem to have higher heart rate (97 vs. 93, p = 0.006) but lower systolic blood pressure (129 vs. 131 mmHg, p = 0.043) and PaO_2_/FiO_2_ (169 vs. 186, p = 0.013). Moreover, patients with CVD were more likely to have higher neutrophil (8.47 vs. 7.69 × 10^9^/L, p = 0.015), troponin T (30.4 vs. 27.45 ng/mL, p = 0.023) and brain natriuretic peptide (1195.0 vs. 1037.5 pg/mL, p = 0.048) but lower lymphocyte (0.78 vs. 0.85 × 10^9^/L, p = 0.026) and albumin (31.2 vs. 32.4 g/L, p = 0.005) than that in non-CVD patients.

**Fig. 2:**
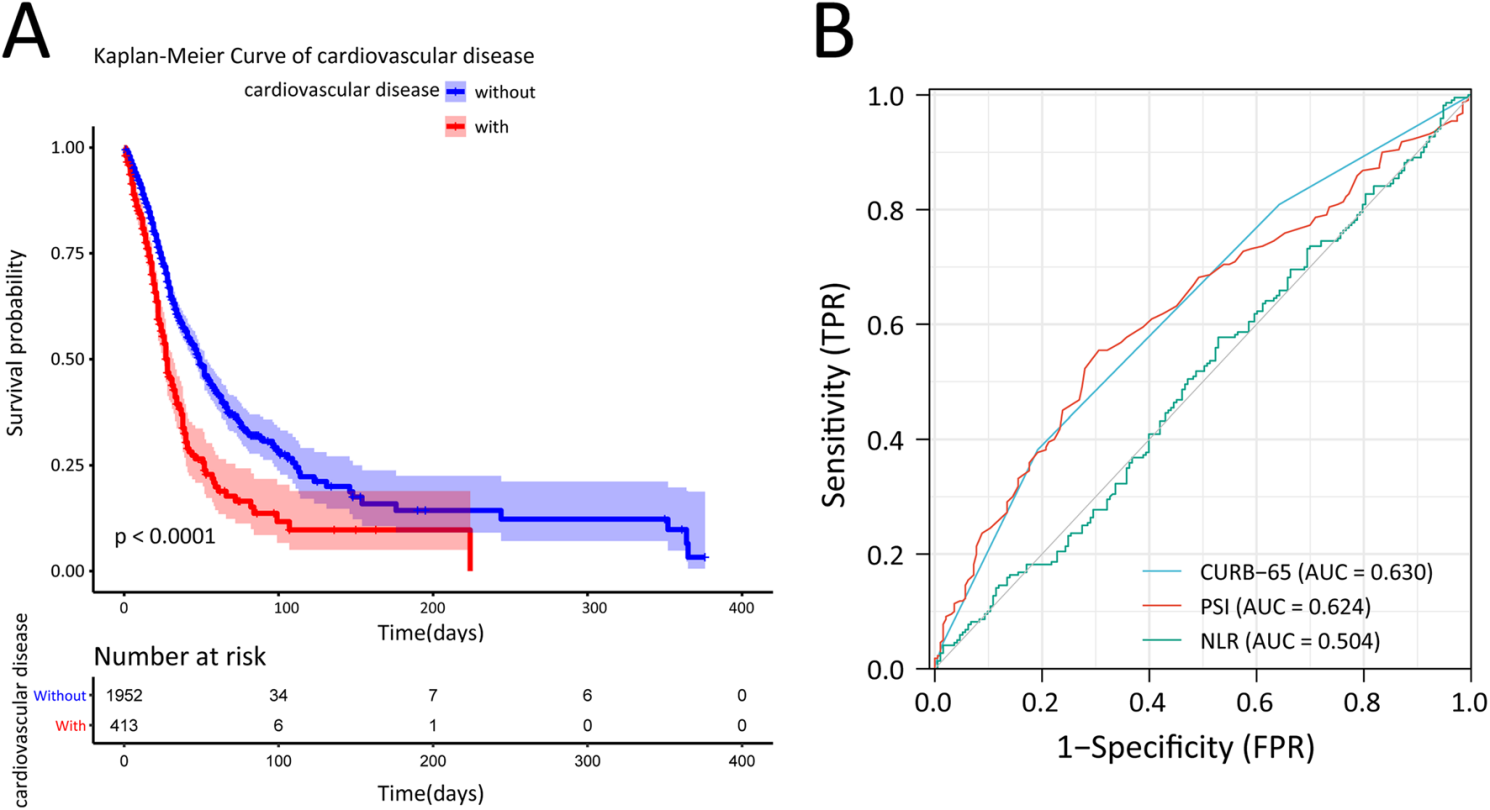
Prediction of in-hospital mortality in elderly SCAP patients with CVD by clinical scores. **A** Survival curves of SCAP patients with or without CVD. **B** ROC analysis for CURB-65, PSI and NLR. The AUC was 0.630 and 0.624 respectively. *SCAP* severe community acquired pneumonia, *CVD* cardiovascular disease, *ROC* receiver operating characteristic, *AUC* area under the curve

MACE such as myocardial infraction (MI), arrhythmia, cardiac failure and stroke are considered to be an important cause of poor prognosis and cardiac death in patients with CVD [12]. However, in the present study, only MI was found to have adverse impacts on the prognosis of elderly SCAP patients with comorbid CVD (3.9 vs. 1.6%, p = 0.003). The incidences of arrhythmia, cardiac failure and stroke were not significantly different between the two groups during hospitalization. Notably, substantial differences in clinical outcomes were observed between the two groups. Compared with patients without CVD, patients with CVD had a higher 7-day mortality (12.1 vs. 5.8%, p < 0.001), 14-day mortality (21.3 vs. 12.0%, P < 0.001), 28-day mortality (41.6 vs. 23.6%, P < 0.001), ICU mortality (47.2 vs. 28.7%, P < 0.001), and hospital mortality (53.3 vs. 33.8%, p < 0.001) (Fig. 2A). A prospective clinical study indicated that the neutrophil-to-lymphocyte ratio (NLR) showed an ideal prognostic value in elderly adults with CAP [13]. Nevertheless, the NLR (0.504; 95% CI 0.448, 0.560), as well as the two most widely used severity assessment tools in SCAP, PSI (0.624; 95% CI 0.521, 0.631) and CURB-65 (0.630; 95% CI 0.576, 0.683) scores, were poor predictors of in-hospital mortality in elderly SCAP patients with CVD in the present study (Fig. 2B). Thus, it is urgent to find an effective prognostic evaluation model in this population.

### Development of nomogram

**Fig. 3:**
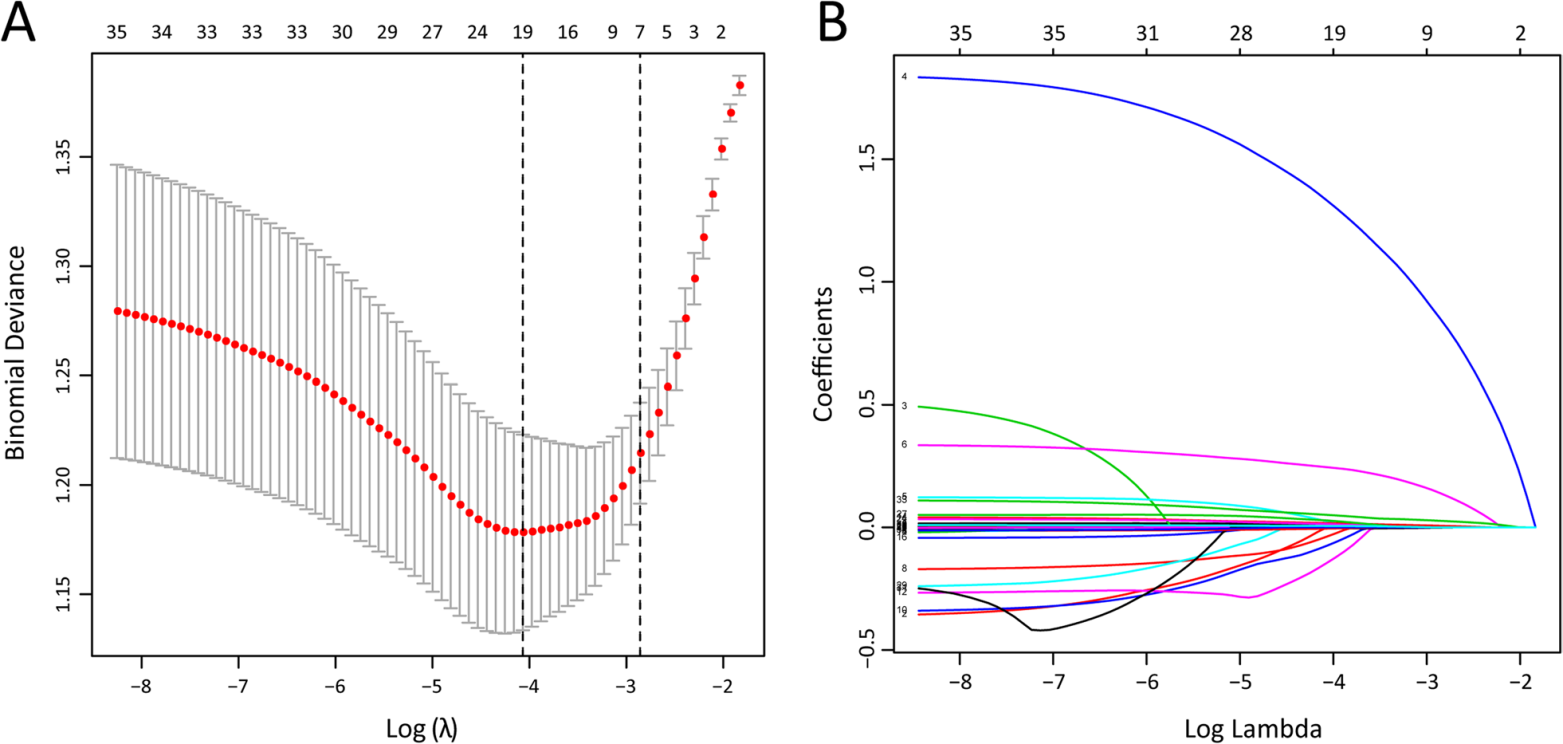
Potential risk factors were selected using the LASSO binary logistic regression model. **A** Fivefold cross-validation was used to select optimal parameter (lambda) in the LASSO model via minimum criteria. **B** LASSO coefficients profiles of 19 clinical features with non-zero coefficients determined by the lambda. *LASSO* least absolute shrinkage and selection operator

**Fig. 4:**
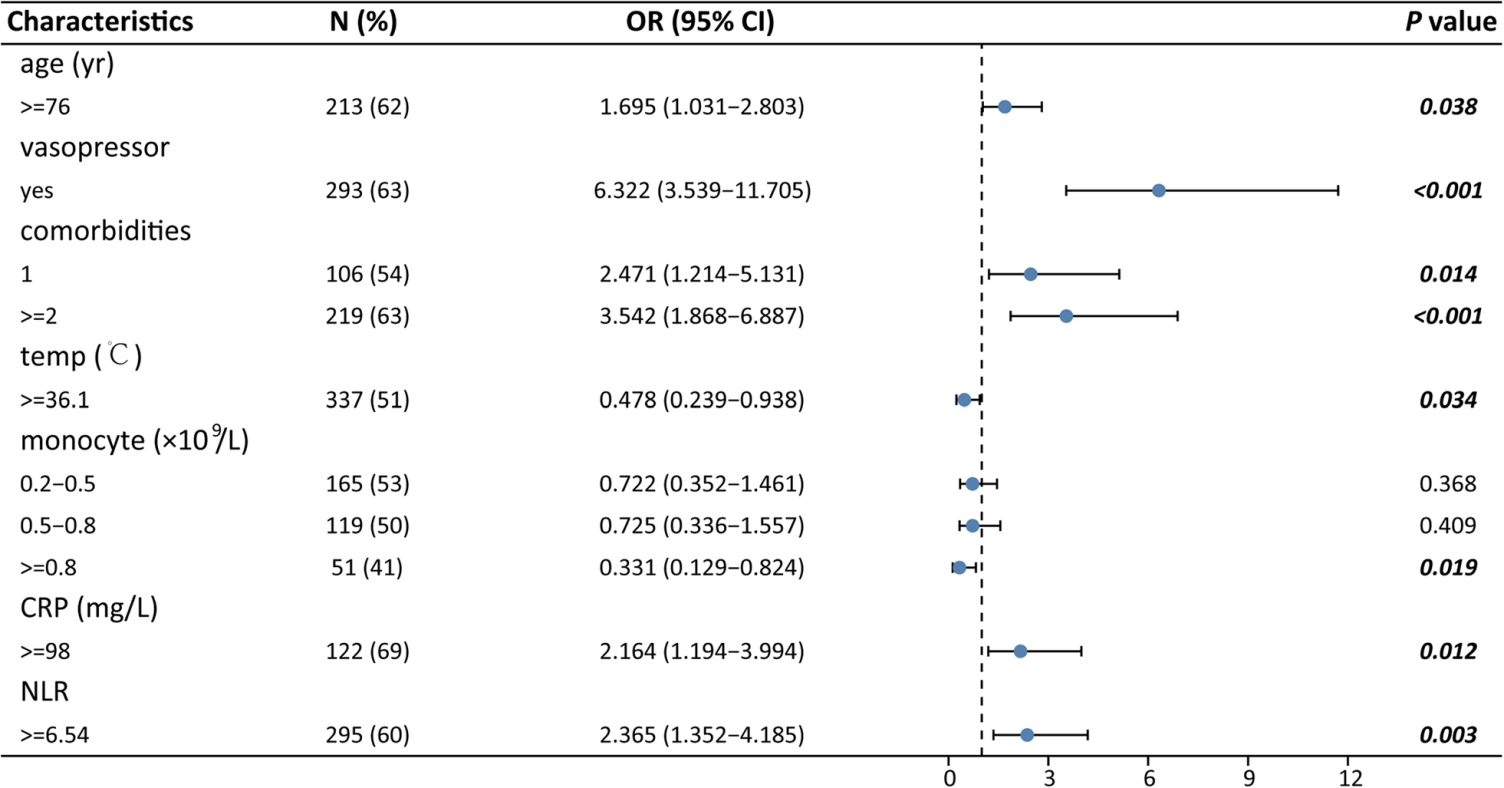
Independent risk factors for hospital mortality of elderly SCAP patients with CVD. *OR* odds ratio, *SCAP* severe community acquired pneumonia, *CVD* cardiovascular disease

A total of 23 variables were found to be potential risk factors for in-hospital mortality in the univariate logistic regression analysis (Table 2). Next, the logistic LASSO regression model was performed to select the vital prognostic predictors from all clinical characteristics with in the elderly SCAP patient cohort (see “Materials and Methods” for details). The preliminarily screened clinical signatures consisting of 19 potential features including age, disturbance of consciousness, the use of vasopressor, numbers of primary symptoms, body temperature, heart rate, albumin, monocyte, hemoglobin, APTT, aspartate transaminase (AST), brain natriuretic peptide (BNP), uric acid (UA), blood glucose, C-reactive protein (CRP), lactic acid, total bilirubin, blood urea nitrogen (BUN) and NLR (Fig. 3). Then, these variables were rescreened using multivariate logistic regression and only 7 features were identified as independent prognostic factors for hospital mortality: age, the use of vasopressor, numbers of primary symptoms, body temperature, monocyte, CRP and NLR. Their ORs and 95% CI are shown in Fig. 4.

**Fig. 5:**
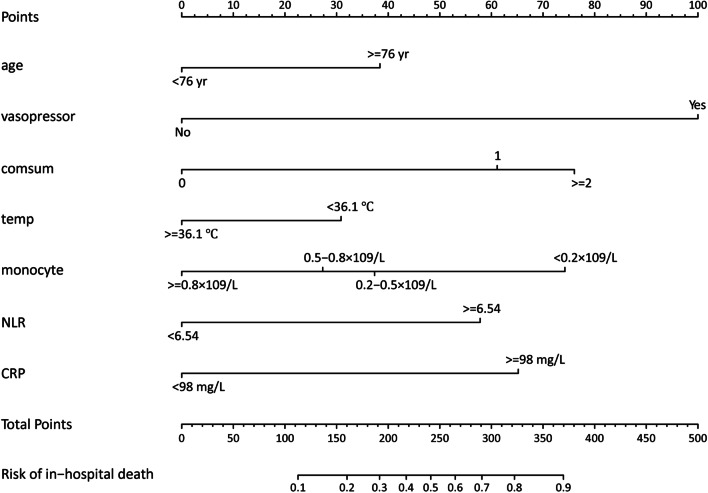
The nomogram for hospital mortality in older SCAP patients with CVD. *CRP* C-reactive protein (mg/L), *NLR* neutrophil to lymphocyte ratio

As illustrate in Fig. 5, the selected 7 risk factors were used to construct an intuitive nomogram for predicting the in-hospital mortality in elderly SCAP patients with CVDs. Each predictive factor was assigned a corresponding score, which was displayed on the top line of the nomogram. The total point was assessed by summing each score from these 7 selected features, and the location of the total score was vertically projected onto the predictor axis, thus obtaining an individualized risk estimation of mortality in hospitalized elderly SCAP patients with CVD.

### Validation and calibration of nomogram

**Fig. 6:**
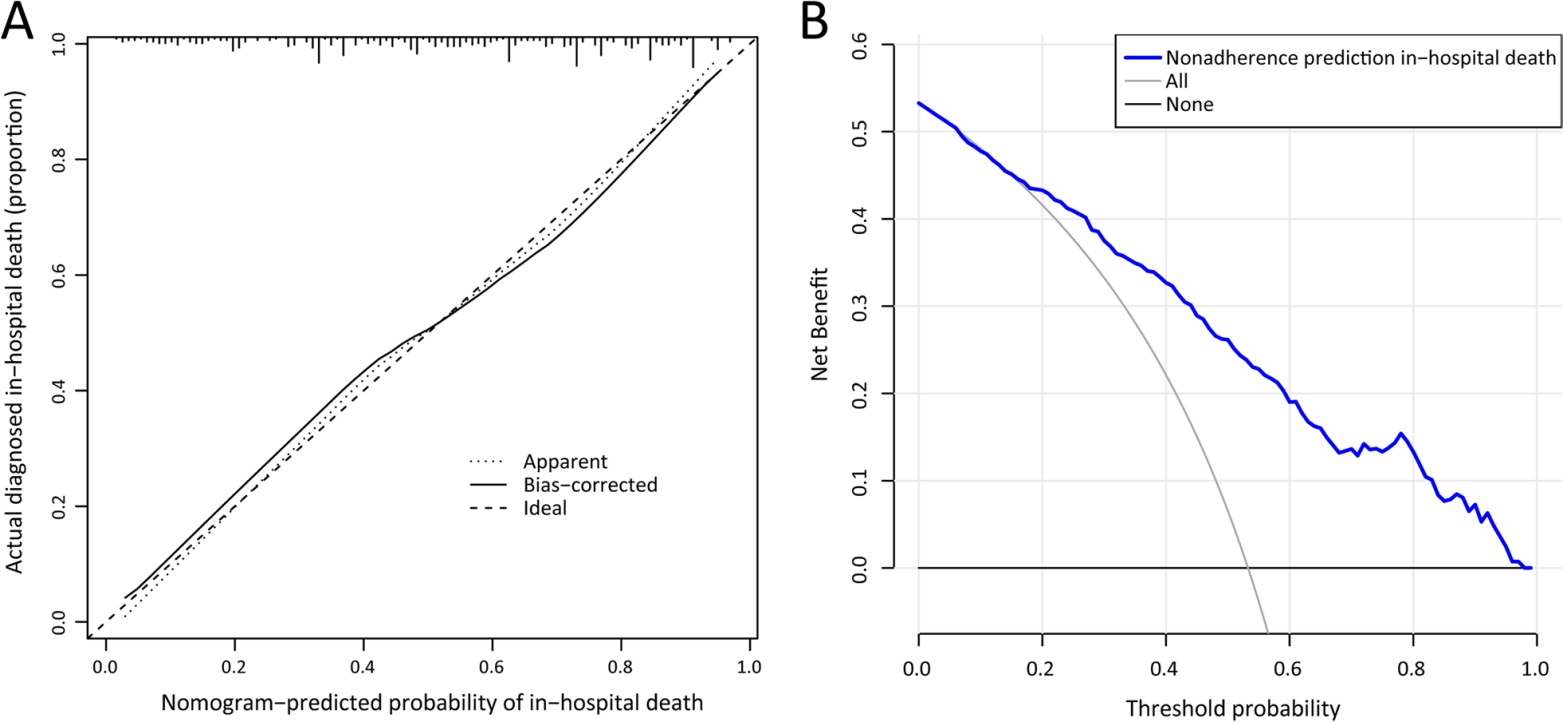
Calibration curve (**A**) and DCA (**B**) of nomogram. *DCA* decision curve analysis

The C-index for the constructed prediction model was 0.800 (95% CI 0.758–0.842), and was verified to be 0.781 through bootstrapping validation. It demonstrated that our model holds a better prediction accuracy for predicting the in-hospital mortality in elderly SCAP patients with CVDs, compared with PSI (0.624; 95% CI 0.521, 0.631) and CURB65 (0.630; 95% CI 0.576, 0.683) scores. Moreover, the C-index (0.711; 95% CI 0.687–0.735) of this nomogram in patients without CVD was also calculated, which was quite lower than that in patients with CVD. In Fig. 6A, the calibration curve indicative a good consistency between the monogram predicted risk and the actually observed risk, as the calibration curve was quite close to the reference line. Whereafter, the decision curve manifested that the nomogram had good overall net benefits within a wide range of threshold probabilities, demonstrating the clinical usefulness of our nomogram (Fig. 6B).

## Discussions

SCAP is a life-threatening disease which contributes to be the major cause of infection-associated death worldwide [5]. Failure to provide timely diagnosis, assessment of the severity of CAP and initiation of appropriate treatment could result in water, acid-base and electrolyte balance disorders, then leading to multiple organ dysfunction [14]. Epidemiological investigation shows the incidence of SCAP tends to increase markedly with age (persons aged 65 years or older) [1, 5]. Especially in developed countries, it has been reported that almost half of the hospitalizations for pneumonia occurs in patients over 65 years [15]. In our study population, SCAP patients older than 65 years accounted for about 43%. Nowadays, an accumulation of evidences indicated that elderly patients showed heavier clinical manifestations and worse outcomes compared with younger ones [16]. Poor clinical prognosis of elderly SCAP patients may be associated with multiple comorbidities, rather than with multidrug-resistant bacteria [5]. Based on these findings, our present study focused on elderly SACP patients and explored the impact of CVD, a common comorbid disease in the aged on their prognosis [17].

Strong research evidence linking CVD with advancing age; however, how it drives disease progression in elderly SCAP patients is still unclear [18]. Consistent with previous studies, our research found that the CVD cohort had a significant higher risk of in-hospital mortality than the non-CVD cohort did (53.3 vs. 33.8%, p < 0.001) [19, 20]. Besides, compared with elderly SCAP patients without CVD, those who had comorbid CVD were more likely to show cardiopulmonary symptoms, such as cough, sputum and chest pain. This may be the results of that patients with chronic CVD usually combined with cor pulmonale, or were more likely to develop it [21]. Meanwhile, pneumonia has also been confirmed to be an independent risk factor for inducing or aggravating cor pulmonale [22]. Although a bulk of studies showed that biomarkers such as CRP, PCT, proadrenomedullin, cytokines and others could provide additional information about SCAP prognosis [23–25]. It should be recognized that some of these biomarkers may not be applicable in elderly patients with SCAP, because older patients tend to be less sensitive and have nonspecific clinical manifestations as they age [26, 27]. Our study observed that the levels of TnT and BNP in CVD group were slightly higher than the control group; whereas, the levels of those two indicators were much higher than the high limit of normal value in both groups, revealing a comparable degree of cardiovascular dysfunction. This occurs mostly on account of the cardiovascular damage, which caused by the infection and the inflammatory host response, actually takes place in the majority of elderly SCAP patients [26]. Thereby, the leaving sequelae is predisposed to the occurrence of MACE and mortality [28].

Few studies have assessed the risk factors for hospital mortality in elderly SCAP patients with CVDs; our retrospective analysis demonstrated that in-hospital mortality in this group of patients was more than 50%, much higher than in patients without CVDs (33.8%, p < 0.001). A growing number of literatures documented that MACE more frequently appeared in patients with previous CVDs, or SCAP episodes without known previous cardiovascular disease (around 15–30%) [3, 9, 19]. Moreover, the incidence of heart disease is greatest among middle-aged and elderly persons, populations that are also at the greatest risk for pneumonia [29]. Our data revealed that there was no significant difference in MACE among elderly SCAP patients with or without chronic CVDs except for MI. According to these findings, in elderly SCAP patients with coexisting CVDs, the occurrence of MACE may not be the most important risk factor for mortality. Further data analysis also supported our hypothesis. After the LASSO and multivariate logistic regression analysis, only 7 independent risk factors were selected including: age, the use of vasopressor, numbers of primary symptoms, body temperature, monocyte, CRP and NLR, Lacking of specific indicators of myocardial injury. These suggested that in patients with very poor baseline conditions (old age, co-CVDs, and SCAP), hypothermia, monocytopenia or elevated levels of acute inflammatory markers (NLR and CRP) represented a poor prognosis [30]. Anyhow, our results indicated that elderly SCAP patients with CVDs have significantly increased mortality and relatively severe myocardial injury. Therefore, evaluation of the risk factors for death in this population will help us identify truly high-risk patients at an early stage, as well as select appropriate treatment strategies.

Assessment of the severity is quite crucial for the initial evaluation of CAP patients [3, 31], and the PSI and CURB-65 are the most frequently used ones [5]. Some studies have revealed that these disease-specific scores are less sensitive in the elderly patients compared to younger adults [6, 32]. When evaluating the performance of PSI and CURB-65 scores to predict in-hospital mortality in SCAP patients aged ≥ 80 years via the receiver operating characteristics curve, the area under the curves (AUCs) were 0.61 (95% CI 0.51–0.71) for the CURB-65 and 0.52 (95% CI 0.41–0.62) for the PSI respectively [6]. Lv et al. found that CURB-65 score did not exhibit superiority in predicting hospital mortality of patients aged 65-years and older with CAP [33]. Similar to previous studies, our research demonstrated that both PSI and CURB-65 did not reveal well predictive of in-hospital mortality in elderly SCAP patients with CVDs. This reflects that both PSI and CURB-65 scores were less effective in predicting in-hospital mortality of the elderly, albeit performed well in identifying SCAP patients [34, 35].

Cataudella et al. indicated NLR (AUC = 0.94) predicted 30-day mortality in elderly CAP patients better than PSI and CURB-65 score [13]. Besides, researchers also demonstrated NLR was a strong predictor of predicting the presence and the number of carotid atherosclerotic lesions in elderly adults [36]. However, we found that NLR along was not a good index to predict in-hospital mortality of elderly SCAP patients with CVD in the present study. This may be due to the fact that our patients were sicker (all were SCAP) and the inflammation was relatively suppressed. Moreover, the inclusion and exclusion criteria of our study are quite different from those of these studies, which may lead to a large difference in the study population, so the prediction efficiency of NLR decreases. Therefore, we constructed a specific and simple prediction model based on the characteristics of this population with a promising C-index of 0.800 (95% CI 0.758–0.842), which was also confirmed to be 0.781 (the corrected C-index) via bootstrapping validation. The selected predictive factors which developed the nomogram were all from the most common blood tests and physical examination, further improving its practicability and operability. We also tested the predictive power of nomogram in elderly patients without CVD, and found the C-index was only 0.711 (95% CI 0.687–0.735), suggesting that the variables screened into nomogram were more specific to population of elderly SCAP patients with CVD, such as NLR.

Despite the strengths mentioned above, there were inevitably several limitations to our study should be addressed. First, this was a retrospective study conducted in a single center from a large tertiary hospital in China. Although the robustness of the nomogram was examined with internal validation, the enrolled population seemed to be not abundant. Therefore, whether it can be extended to other hospitals in China or even in a Western population needs to be externally evaluated in wider SCAP populations. Second, the therapeutic strategies were varied from patient to patient. In addition, data collected retrospectively may be biased, which may have influenced the accuracy of the nomogram model. Prospective multi-center studies that can ensure generalizability and the collection of accurate data are needed. Third, risk factor analysis did not include all potential factors that affected the mortality in hospitalized SCAP patients. Some valuable predictors were not thoroughly assessed such as the social support, the species of pathogenic bacteria, antibiotics [37, 38] and other factors. Lastly, we used the in-hospital mortality in Kaplan–Meier survival curves, but long-term outcomes were not recorded.

## Conclusions

In conclusion, the present study was performed to explore the correlations between CVDs and outcomes of elderly SCAP patients. Moreover, we presented an easily applied nomogram and can predict in-hospital mortality in elderly SCAP patients with comorbid CVDs. The C-index of nomogram indicated a high prediction accuracy of our model, which suggested that our nomogram is promising and deserves to be further explored in the future. With the assessment of individual risk, clinicians can take more necessary measures on medical interventions or a more reasonable protocol of therapy, which may help to improve the treatment outcome.

## Data Availability

The datasets used and/or analyzed during the current study are available from the corresponding author on reasonable request.
